# Apparent soil electrical conductivity and gamma-ray spectrometry to map particle size fraction in micro-irrigated citrus orchards in California

**DOI:** 10.3389/fpls.2025.1512598

**Published:** 2025-03-10

**Authors:** Elia Scudiero, Michael P. Schmidt, Todd H. Skaggs, Jorge F. S. Ferreira, Daniele Zaccaria, Alireza Pourreza, Dennis L. Corwin

**Affiliations:** ^1^ Environmental Sciences Department, University of California, Riverside, Riverside, CA, United States; ^2^ U.S. Salinity Laboratory, United States Department of Agriculture (USDA) – Agriculture Research Service, Riverside, CA, United States; ^3^ Department of Land, Air and Water Resources, University of California, Davis, Davis, CA, United States; ^4^ Biological and Agricultural Engineering Department, University of California, Davis, Davis, CA, United States

**Keywords:** particle size fraction, precision agriculture, apparent electrical conductivity, gamma-ray spectrometry, near-ground sensing

## Abstract

In specialty crops, water and nutrient management may be optimized using accurate, high-resolution soil maps, especially in resource-constrained farmland, such as California. We evaluated the use of soil apparent electrical conductivity (EC_a_) and gamma-ray spectrometry (GRS) to map particle size fraction across three micro-irrigated non-saline citrus orchards in California. Our research showed that EC_a_ was a reliable predictor of soil texture, particularly sand and silt contents, with Pearson correlation coefficients (*r*) as high as -0.92 and 0.94, respectively, at the field level. Locally-adjusted analysis of covariance (ANOCOVA) regressions using EC_a_ data returned accurate sand, silt, and clay content estimations with mean absolute errors (MAE) below 0.06, even when calibrated with a limited dataset (n=5 per field). On the other hand, we observed mixed results with GRS. We observed negative correlations between GRS total counts and sand content over the entire dataset (*r* = -0.55). However, one site (Strathmore) showed a field-scale positive correlation (*r* = 0.88). Clay content significantly correlated with gamma-ray total counts (TC) over the entire dataset (*r* = 0.37) but not at the field scale. Additional soil data analyses using GRS radionuclide ratios and soil laboratory analyses using diffuse reflectance infrared Fourier transform spectroscopy and acid ammonium oxalate extractable elements indicated unique geochemical and mineralogical characteristics in Strathmore, suggesting that factors such as soil mineralogy influenced the GRS measurements. This inconsistency prevented the development of a multi-field GRS-based soil texture ANOCOVA model. These findings confirm that EC_a_ is highly effective for soil texture mapping in non-saline soils using linear modeling, while GRS may require field-specific calibration due to variations in local mineralogy. Integrating multi-sensor data is a viable means for reducing ground-truthing requirements and related costs, and improving the quality and accuracy of soil maps in agriculture.

## 1 Introduction

Accurate soil maps are critical for efficient and sustainable nitrogen and water management in specialty crops like citrus. Variations in soil properties, such as texture and moisture content, remarkably influence the availability and uptake of nutrients and water. Soil texture, determined by the proportion of sand, silt, and clay particles, affects key soil processes such as water retention, drainage, and nutrient-holding capacity. These processes, in turn, influence the movement and availability of water-soluble nitrogen and other macro and micro elements applied as fertilizers and diffused within the soil profile. Nitrogen-deficient citrus plants are stunted, whereas excessive nitrogen promotes vegetative growth and increases susceptibility to diseases that damage fruit, kill spurs, and may reduce yields in following years (Muhammad et al., 2018). Timing of nitrogen fertilization is crucial in citrus. Peak nitrogen uptake in citrus trees happens during blooming and early fruit growth (Muhammad et al., 2018). In areas with coarse-textured soils (e.g., sandy soils), the high permeability and low water-retention capacity may lead to nitrogen leaching, particularly in the form of nitrate (NO_3_^-^), beyond the root zone and into groundwater. Nutrient leaching reduces the fertilizer use efficiency by crops and increases the risk of groundwater contamination, contributing to issues such as eutrophication of water bodies and pollution of drinking water sources. Conversely, in fine-textured soils (e.g., clay-rich soils), higher water retention and slower drainage may lead to waterlogged conditions, especially in low-lying areas. Under such anaerobic conditions, denitrification processes may dominate, converting nitrate into gaseous forms of nitrogen such as nitrous oxide (N_2_O), a potent greenhouse gas. Fields characterized by wide variability of soil texture and terrain attributes may have contrasting propensities for nitrogen loss through leaching or denitrification (Luo et al., 2023). Without accurate mapping and management of these variations, uniform application prescriptions of nitrogen and other nutrients can result in over-application in some areas and under-application in others. Over-application can intensify nitrogen losses and environmental impacts, especially in coarser soil (Syvertsen and Smith, 1996; Muhammad et al., 2018; Luo et al., 2023).

In the United States of America, soil survey maps developed by the government (e.g., USDA-NRCS soil survey maps) provide valuable broad-scale information on soil types and properties (Beaudette and O’geen, 2009; Chaney et al., 2016). Still, they are generally inadequate for guiding detailed field-scale water and nutrient management (Scudiero et al., 2024b). These maps, typically created at scales of 1:12,000 to 1:24,000, offer an overview of soil variability across landscapes but often fail to capture the fine-scale heterogeneity within individual fields, which is critical for precision agriculture (Reyes et al., 2018).

On-the-go soil sensing technologies provide an efficient means to generate high-resolution soil maps across agricultural fields. Among these technologies, apparent soil electrical conductivity (EC_a_), gamma-ray spectrometry (GRS), and other on-the-go near-ground sensing technologies have been widely studied for their potential to map soil texture (Sudduth et al., 2005; Pätzold et al., 2020).

Apparent soil electrical conductivity (EC_a_) is a measure of how easily electrical current passes through the soil, influenced by factors such as soil texture, moisture content, salinity, and temperature (Rhoades et al., 1976; Corwin and Lesch, 2003). EC_a_ is commonly used in precision agriculture to map soil variability, as it provides indirect information about soil properties that affect crop growth, such as clay content and water-holding capacity (Corwin and Lesch, 2003). This non-invasive, on-the-go sensing method is valuable for identifying zones within a field that require different management practices (Córdoba et al., 2016). Geospatial EC_a_ is arguably the most used sensor measurement for field-scale soil mapping by practitioners and scientists due to its relative ease of use, cost-effectiveness, and ability to capture spatial variability in soil properties influenced by factors such as moisture content, salinity, and clay content (Corwin and Lesch, 2003; Doolittle and Brevik, 2014). However, in environments where soil salinity is expected, such as in California and other mediterranean, arid, and semi-arid irrigated farmland worldwide, EC_a_ may not be the ideal tool for soil texture mapping because of salinity becoming a primary factor influencing the EC_a_ measurement (Corwin and Scudiero, 2016). Notably, EC_a_ measurements should be carried out over moist soils (i.e., around field capacity or slightly drier) to ensure reliable correlations with target soil properties (Corwin and Lesch, 2005b). To this regard, micro-irrigated orchards in water-scarce environments present unique challenges for EC_a_ sensing due to the very-short scale spatial heterogeneity of wetting soil conditions, which influences the sensor measurements. Soil moisture levels are generally ideal for reliable EC_a_ surveys in the hardly accessible areas under dense canopies, where micro irrigation is applied. Conversely, easily accessible alleyways have generally much drier soils (Pedrera-Parrilla et al., 2016; Corwin et al., 2022; Scudiero et al., 2024a). Soil compaction may also be remarkably different between below-canopy areas and the alleyways due to field equipment passages and other traffic. Even after precipitation events, EC_a_ surveys in the alleyways of micro-irrigated orchards may potentially lead to biased representation of field-scale soil spatial variability. Corwin and Lesch (2013) recommend surveying EC_a_ both in alleyways and along the tree lines distinctively in orchards and vineyards.

Gamma-ray spectrometry (GRS) measures, non-invasively, the natural gamma radiation emitted by isotopes of potassium (K), uranium (U), and thorium (Th) present in the soil. The entire energy spectrum of the gamma radiation, typically around the 0.1 to 3 MeV range, is also measured as Total Counts (TC) of gamma emission. Gamma-ray emitting nuclides are naturally present in soils and rocks. At the field scale, these gamma emissions correlate with specific soil properties, such as texture and mineral composition. Some factors influence gamma-ray emissions, e.g., increasing water content and bulk density decrease the measured gamma-ray volume (Grasty, 1979; Cook et al., 1996; Reinhardt and Herrmann, 2019). Mahmood et al. (2013) and Reinhardt and Herrmann (2019) provided detailed descriptions of GRS and its use for soil mapping and precision agriculture. Unlike EC_a_, which primarily reflects soil moisture and salinity, GRS provides information on the mineralogical composition of the soil, offering a different perspective on soil heterogeneity. Moreover, it is recommended that EC_a_ measurements are carried out in moist soils (Corwin and Lesch, 2005b), whereas GRS is attenuated by high soil moisture (Reinhardt and Herrmann, 2019). In water-scarce and dry micro-irrigated orchards, where spatial variability in soil moisture is extremely short-scaled due to localized irrigation application under the tree canopies, GRS can complement EC_a_ taken along the driplines by providing additional data on particle size distribution and mineral content with measurements done in the drier alleyways (Scudiero et al., 2024a). This dual-sensor approach, developed and described by Scudiero et al. (2024a), can enhance the accuracy of soil maps, enabling more precise water and nutrient management in these complex environments.

Developing field-scale models from sensor data typically requires extensive ground-truthing (Reyes et al., 2018), involving the collection of numerous soil samples to calibrate and validate the sensor measurements (Corwin and Lesch, 2005b), as otherwise sensor maps only serve as a qualitative indication of soil spatial variability (Corwin and Scudiero, 2016). For regional-scale models, two primary approaches can be utilized: universal models, which apply broadly across regions (Lobell et al., 2010; Pätzold et al., 2020), and locally adjusted models, such as analysis of covariance (ANOCOVA) regression models (Corwin and Lesch, 2014), which tailor the sensor data to specific local conditions. The choice between these approaches depends on the degree of variability within the region, the desired accuracy of the soil maps, and the available resources that can be used for the ground truthing campaign. The reliability of soil property models, whether universal or locally adjusted, is highly dependent on the rigor and consistency of sensor and soil data collection protocols (Corwin and Lesch, 2005b). Standardized calibration, maintenance, and handling of sensors are essential to ensure accurate and comparable measurements across different locations and times. Proper spatial sampling design and timing are critical to capturing the full range of soil variability (Lesch, 2005), while standardized soil sampling and laboratory procedures ensure the accuracy of ground-truthing data (Corwin and Yemoto, 2017). Consistent adherence to these protocols minimizes measurement-induced variability, leading to more reliable and generalizable soil property models (Corwin and Scudiero, 2016). Protocols for field to regional scale soil mapping with EC_a_ and for EC_a_-directed soil sampling have been developed and updated by Dennis Corwin and colleagues at the USDA-ARS US Salinity Lab (Corwin and Lesch, 2005b; Corwin and Scudiero, 2016). However, no equivalent protocols or recommendations for GRS are available (Reinhardt and Herrmann, 2019).

Universal models, also referred as “site-independent models” (Pätzold et al., 2020) should predict soil properties at novel agricultural fields without the need for additional ground truthing. For these models to be reliable in predicting soil properties across a broad region, several conditions must be met. There should be a quantifiable mechanistic relationship between the sensor measurements and the target soil properties across the entire region. This requires that the physical processes being measured, such as electrical conductivity or gamma-ray emissions, correlate strongly and predictably with soil attributes like texture, moisture content, or mineral composition, regardless of local variations in soil type or environmental conditions. The data used to develop the model should be representative of the full range of conditions within the region, ensuring that the model is not biased toward specific soil types or microclimates. Modeling approaches like support vector machines were shown as good candidates for predicting soil properties from on-the-go sensor measurements over datasets with diverse pedogenesis (Heggemann et al., 2017). Additionally, the secondary factors influencing the sensor measurements, e.g., soil-forming processes, land use history, tillage, and other factors (Corwin and Lesch, 2005b; Reinhardt and Herrmann, 2019), are similar enough across the region that a single model can adequately describe the soil property-sensor relationships everywhere. Finally, to confirm its accuracy and generalizability, a universal model should be validated against independent data sets from different locations within the region (Ramcharan et al., 2018) and with robust cross-validation techniques (Roberts et al., 2017).

Locally adjusted models, such as the ANOCOVA method operate under the assumptions that sensors consistently measure physical processes related to the target soil property across a given geographical regions and that any secondary influencing factors can be accounted for by adding local ground-truth data any time a new site is surveyed. If this assumption holds true, in ANOCOVA models, a constant slope can be applied to the model, allowing for the estimation of a local random effect (i.e., a field-specific intercept coefficient) with limited soil sampling (Harvey and Morgan, 2009; Corwin and Lesch, 2014, 2017; Scudiero et al., 2017). This approach potentially reduces the need for extensive ground-truthing while still providing accurate soil property estimates at the regional scale (Scudiero et al., 2017). In the context of calibrating on-the-go sensor measurements to map soil texture, ANOCOVA regression relies on several assumptions to ensure accurate results. First, ANOCOVA assumes a linear relationship between the sensor measurements (covariate) and the soil texture properties (dependent variable) across the region of interest, with the slope of this relationship being consistent across different conditions. Any interaction between sensor data and geographical or management differences should not significantly alter the slope of the relationship for the ANOCOVA model to be reliable. Second, ANOCOVA assumes that the residuals, or errors, of the regression model are normally distributed and exhibit homoscedasticity, meaning that the variability of these errors is consistent across all levels of the covariate. Moreover, the model assumes that the covariate is measured without error, which is critical for the reliability of the calibration process. For this, it is recommended that the sensor data collection procedure is methodologically consistent across the entire dataset (Corwin and Lesch, 2014).

The objective of this study was to evaluate the use of GRS for mapping particle size fraction in micro-irrigated citrus orchards in California and to compare its performance to that of EC_a_, which served as the benchmark. Additionally, the study aimed to test whether the ANOCOVA approach could be effectively applied to GRS data to develop accurate soil maps with reduced ground-truthing requirements.

## 2 Materials and methods

### 2.1 Research sites

**Figure 1:**
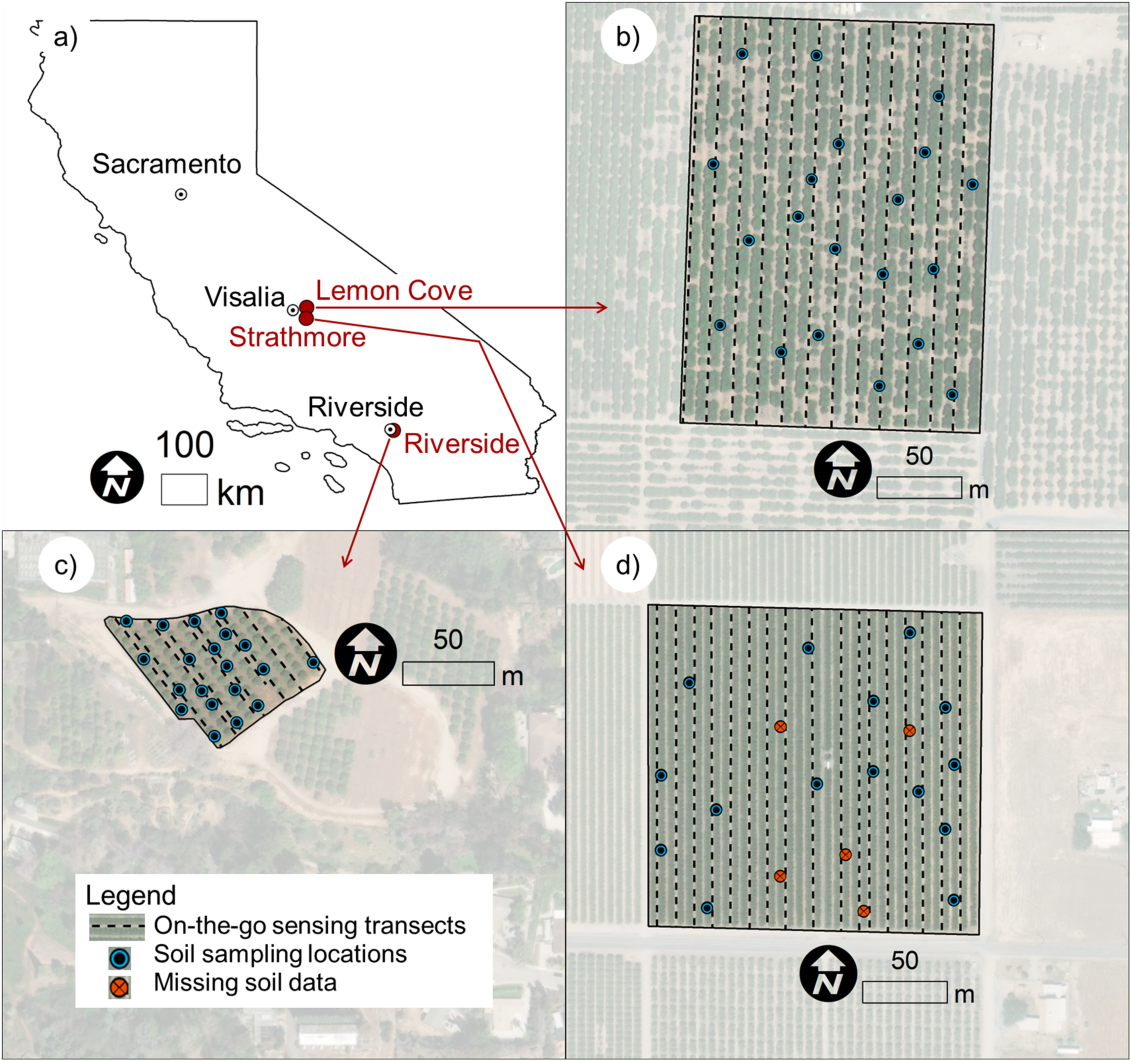
**(a)** the three research sites located in California, USA; **(b)** the 4.2-ha ‘Navel’ orange orchard located in Lemon Cove; **(c)** the 0.4-ha ‘Navel’ orange orchard located in Riverside; and **(d)** the 3.7-ha ‘Page’ mandarin. The location of the on-the-go soil sensing transects, the soil sampling locations, and the missing soil data locations (at the Strathmore site only) are depicted in the figure.

Soils at three micro-irrigated citrus orchards in California, USA, were investigated in this research (**Figure 1**). The sites are named in this manuscript after their location. A 4.2-ha commercial ‘Navel’ orange (*Citrus sinensis* L.) orchard site was in Lemon Cove, Tulare County (**Figure 1b**). A 0.4-ha ‘Navel’ orange orchard was located at the University of California, Riverside Agricultural Experimental Station, Riverside, Riverside County (**Figure 1c**). A 3.7-ha commercial ‘Page’ mandarin (*Citrus reticulata* B.) orchard located in Strathmore, Tulare County (**Figure 1d**). Trees at the ‘Navel’ orange sites were planted on flat terrain, while trees at the ‘Page’ mandarin site were planted on 0.3-m raised berms made with local soils.

The primary soil types (USDA soil series) were retrieved from Soil-Web (Beaudette and O’geen, 2009): in Lemon Cove they were Havala loam and Yettem sandy loam; in Riverside they were Monserate sandy loam; and in Strathmore they were Porterville clay, San Joaquin loam, and a portion of the field was classified as “Riverwash”.

### 2.2 Sensor-directed spatial sampling scheme delineation and soil sampling

**Figure 2:**
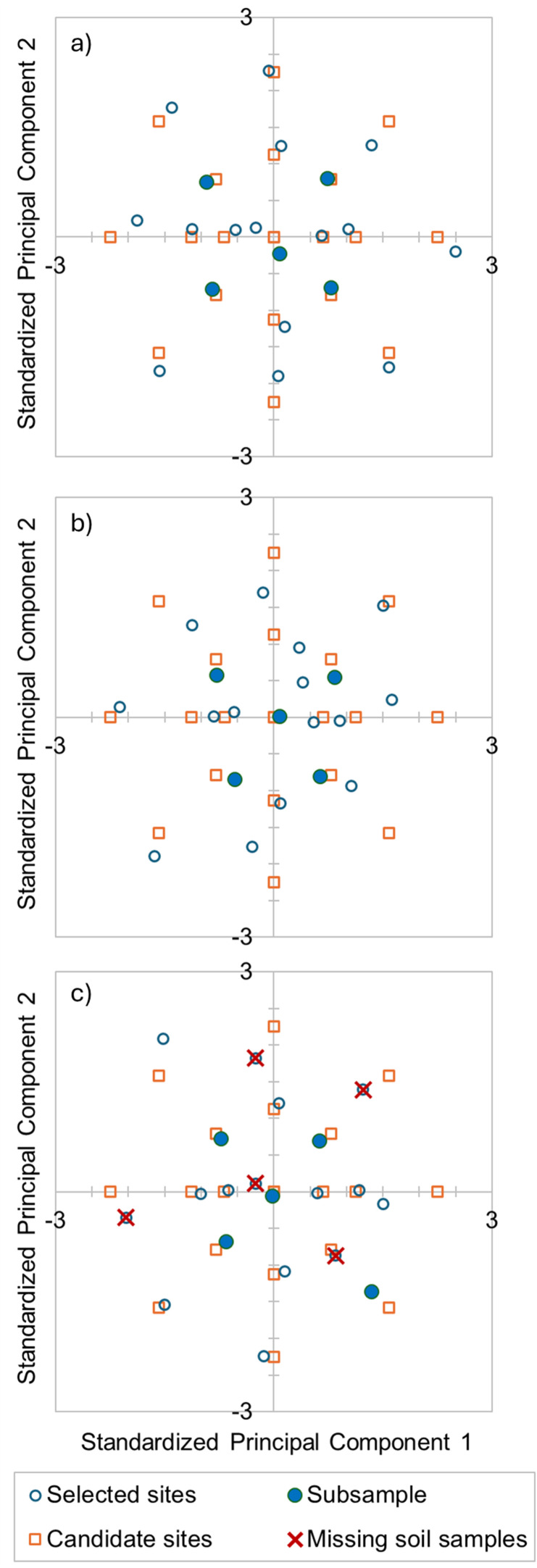
Plots of standardized principal component target response surface sampling design levels (empty squares) and the optimal sites having good spatial uniformity (i.e., selected sites, empty circles) at the three sites: **(a)** Lemon Cove, **(b)** Riverside, and **(c)** Strathmore. The selected subsamples for the Limited Data regression models and soil mineralogy lab analyses are depicted with full circles. At Strathmore, the missing soil samples are depicted with cross symbols.

At each site, sensor-directed spatial sampling selected 20 sampling locations using Response Surface Sampling Design (RSSD) with the ESAP software (Lesch et al., 2000). The RSSD was used with the assumption that geospatial sensors utilized to direct the sampling would correlate with target soil and plant properties of horticultural interest for on-farm experiments. The RSSD identifies a set of candidate principal component coordinates that are representative for the entire sensor survey (i.e., average and standard deviation of the sample equivalent to the one of the population), then selects samples proximal to these principal-component coordinates that are also geographically sparse (i.e., samples are as far as possible from each other, to reduce the risk of autocorrelated residuals when using ordinary least square linear modeling) (Lesch et al., 1995; Lesch, 2005; Fitzgerald et al., 2006). **Figure 2** shows the candidate RSSD sites and selected sites for the three study orchards.

The data from the Lemon Cove and Strathmore orchards were collected as part of an on-farm experiments to map soil with GRS and EC_a_ and to deepen the understanding of soil physical and chemical properties with citrus leaf nutrient contents and fruit yield and quality. At these study sites, spatial sampling was directed using high-resolution multispectral imagery collected with an unmanned aerial vehicle (UAV). Scudiero et al. (2019) detailed the use of ESAP to direct spatial sampling at the Strathmore site. The same methodology was used in Lemon Cove and is briefly described below.

Data at the Riverside site was collected to evaluate on-the-go soil sensing when EC_a_ is measured under the canopy of the trees (i.e., closer to the micro irrigation emitters) with the apparatus discussed by Scudiero et al. (2024a). At this site, EC_a_ and GRS were used to direct the soil sampling scheme delineation as detailed by Scudiero et al. (2024a) and briefly described below.

#### 2.2.1 Lemon cove

Five-band multispectral (Blue, Green, Red, NIR, and Red Edge) imagery was collected with RedEdge Multispectral Camera from MicaSense Inc. (Seattle, WA, USA) flown at an altitude of 100 m above ground level in February 2019. Based on the methodologies described by Zhang et al. (2021), a polygon system was created to uniquely identify each citrus tree in the orchard. The multispectral imagery underwent radiometric calibration, and the allocated area for each tree was segmented using the generated polygons. Subsequently, each tree was segmented from its background (soil) by applying empirical thresholds on the excess green index (EGI) and normalized difference vegetation index (NDVI), where a binary mask was created by multiplying the binary masks of EGI and NDVI, effectively isolating the canopy pixels from non-canopy areas (Moghimi et al., 2020). Average reflectance was calculated for each tree at each band separately and a feature vector consisting of sample trees (rows) and 5 spectral features (columns) was created. This dataset was transformed into two uncorrelated principal components (collectively representing 94.3% of the dataset variance) using STATISTICA (version 12, StatSoft Inc., Tulsa, OK, USA). The two principal components were used to direct the sampling scheme. The Kolmogorov-Smirnov Two-Sample Test was used to confirm that the multispectral reflectance values from the selected trees (i.e., the sample) were not significantly different from those of the entire orchard (i.e., the population). Additionally, the Average Nearest Neighbor tool in ArcMap (v.10.5.1, ESRI, Redlands, CA) was used to confirm that the selected sampling scheme was geographically sparse. Soil was sampled in December 2019, at 40 cm increments, down to 1.2 m. Only the data for the 0-40 cm soil profile will be discussed in this manuscript, as GRS is primarily sensing topsoil properties.

#### 2.2.2 Riverside

In August 2019, GRS and EC_a_ were measured at the Riverside orchard. The EC_a_ (0-1.5m) survey was carried out using the EM38-DD (Geonics Ltd., Mississauga, Ontario, Canada) paired to a cm-scale Trimble R2 GNSS receiver (Trimble, Inc.; Sunnyvale, CA, USA) at 244 locations. GRS was carried out with an RSX-1 detector and the RS-701 gamma-ray spectrometer (Radiation Solutions Inc.; Mississauga, ON, Canada) using the sensor’s internal GPS at 1563 locations. The GRS total counts (TC) data were interpolated using simple kriging with the Geostatistical Analyst toolbox in ArcMap. The kriging model's leave-one-out cross-validation had R^2^ = 0.75. The kriged TC values at the EC_a_ locations were extracted. The EC_a_ and TC data were then used in ESAP to identify 20 sampling locations. The representativeness and geographic spread of the sampling scheme were tested analogously to the Lemon Cove site. Soil cores from 0-40 cm were collected in August 2019.

#### 2.2.3 Strathmore

The Strathmore sampling scheme was determined using ESAP and multispectral UAV imagery collected in 2019 as described by Scudiero et al. (2019) and analogously to the procedure described in Section 2.2.1. At Strathmore, the two principal components collectively represented 96.5% of the multispectral reflectance dataset variance. Soil was sampled in December 2019, at 40 cm increments, down to 1.2 m. The data for the 0-40 cm soil profile will be discussed in this manuscript. Soil sampling was interrupted because of rainfall. The soil sampling crew could not return to the site to complete the soil sampling due to the COVID-19 pandemic that started in early 2020. Because of this, soil was collected only at 15 out of 20 locations (see **Figure 1d**).

### 2.3 On-the-go soil sensing

The on-the-go soil sensing surveys at the three sites were carried out using the same sensors and according to the field protocols described by Corwin and Lesch (2005b). The GRS sensor was mounted on an all-terrain utility vehicle and the EC_a_ sensor was on a non-metallic sled towed by the same vehicle. All surveys were carried out at speeds slower than 8 km per hour, typically around 6 km per hour.

The CMD Mini Explorer 6L or ME6L (GF Instruments, S.R.O.; Brno, Czech Republic) was used to measure EC_a_ at all sites. The sensor was placed on a sled and connected via cable to the GF Instruments data logging unit (CMD/C), which was in the drivers cabin during operation. The ME6L, in the Hi mode, measures EC_a_ at the nominal depth of 0.3, 0.5, 0.8, 1.1, 1.6, and 2.3 m. At the three sites the upper five EC_a_ measurements showed high positive correlations. The fourth layer, the EC_a_ for the 0-1.1 m soil profile, was used in this research. The Trimble R2 GNSS receiver was paired to the CMD/C. The EC_a_ was estimated at the soil sampling locations using simple kriging. Kriging interpolations were carried out on Normal-Score transformed data, with a first-order trend removal. Kriging details for each orchard are reported in **Table 1**.

**Table 1:** Semivariogram and Kriging cross-validation specification for the soil apparent electrical conductivity at the three research sites.

Site	Date	Raw measurements	Semivariogram				Kriging Cross-Validation
		n	Model	Nugget (%)	Partial sill (%)	Range (m)	R^2^
Lemon Cove	Dec-2019	481	Spherical	12.9	87.1	145.0	0.90
Riverside	Oct-2021	534	Stable	8.8	91.2	7.8	0.84
Strathmore	Dec-2019	633	Stable	5.9	94.1	96.1	0.90

The date of acquisition and the number (n) of sensor measurements are reported. The interpolation cross-validation coefficient of determination (R^2^) is reported.

The GRS was carried out with the RS-701 spectrometer, which detects total counts (TC) in the 0.4 to 2.81 MeV range, and emissions of potassium (K, %), uranium (U, ppm), and thorium (Th, ppm). On-the-go GRS measurements are usually characterized by a high signal-to-noise ratio (Minty, 1997; Viscarra-Rossel et al., 2007). Simple kriging in ArcMap was used to reduce the noise of the TC data, which showed autocorrelated spatial data at the three sites. Kriging interpolations were carried out on Normal Score transformed data, with a first-order trend removal. Kriging details for each orchard are reported in **Table 2**. The kriged TC values were then extracted at the soil sampling locations for further analyses. The K, U, and Th did not consistently show autocorrelated spatial structures and were not, therefore, interpolated with kriging.

**Table 2:** Semivariogram and Kriging cross-validation specification for the gamma-ray Total Counts at the three research sites.

Site	Date	Raw measurements	Semivariogram				Kriging Cross-Validation
		n	Model	Nugget (%)	Partial sill (%)	Range (m)	R^2^
Lemon Cove	Dec-2019	4992	Exponential	37.6	62.4	34.1	0.84
Riverside	Aug-2019	1563	Exponential	36.0	64.0	39.2	0.75
Strathmore	Dec-2019	4483	Exponential	0.5	99.5	49.2	0.71

The date of acquisition and the number (n) of sensor measurements are reported. The interpolation cross-validation coefficient of determination (R^2^) is reported.

### 2.4 Soil laboratory analyses

Gravimetric water content (GWC) at time of sampling was measured for all samples. Soil was dried and sieved discarding particles larger than 2 mm. The soil samples were analyzed in the laboratory to measure saturation percentage (SP) and the saturated paste electrical conductivity (EC_e_) (Corwin and Yemoto, 2017). SP is used as a proxy of particle size fraction, with reported very strong positive correlations with clay content and very strong and negative correlations with sand content. The hydrometer method (Gee and Bauder, 1986) was used to determine particle size fraction.

For each field, five soil samples were selected for diffuse reflectance infrared Fourier-transform spectroscopy (DRIFTS) and acid ammonium oxalate extractable element analyses. These five samples were selected to be a balanced subsample of the RSSD design at each field: one sample was at the average point for the two principal components for the sensors that directed the soil samples, and the other four samples were at symmetrical target standard deviation coordinates; e.g., RSSD level coordinates for PC1 and PC2, respectively: (1) -0.79, -0.79; (2) -0.79, 0.79; (3) 0, 0; (4) 0.79, -0.79; and (5) 0.79, 0.79) (see **Figure 2**).

To analyze the mineralogy of soils across sites, DRIFTS and selective chemical extraction were used to probe aluminiosilicate and active, poorly crystalline mineral components, respectively. The DRIFTS spectra were collected from selected soils that were ball milled and diluted to 10% mass concentration with spectroscopic grade KBr prior to analysis. Spectra were collected on an Invenio -R spectrometer (Bruker Optics Inc., Billerica, MA) using an EasiDiff sampling accessory (Pike Technologies, Madison, WI). Spectra were collected from 4000-400 cm^-1^ with 4 cm^-1^ resolution and represented an average of 256 scans. All spectra were collected against a ground KBr background. After collection, spectra were post-processed by atmospheric compensation, smoothing (Savitzky-Golay, 17 points), baseline correction and min-max normalization. Selective extractions of Fe, Al, Mn, Si, U and Th associated with poorly-crystalline, active oxide components from soils was conducted by previously established methods (Mckeague and Day, 1966). Briefly, these components were selectively extracted through addition of 10 mL of 0.2 M acid ammonium oxalate solution to 0.25 g of ball milled soil in a 35 mL polypropylene centrifuge tube and shaken for 4 h in the dark. Extracts were subsequently purified through centrifugation at 10000 x g for 10 min and gravity filtered through Whatman 42 filter paper prior to inductively coupled plasma optical emission spectrophotometric (ICP-OES) quantification of elements (Optima 8000, Perkin Elmer).

### 2.5 Data analysis

The Pierson correlation coefficients (r) were calculated to investigate the relationships between soil properties and EC_a_ or TC for each orchard and for the entire dataset.

The use of analysis of covariance (ANOCOVA) regression (Corwin and Lesch, 2014) to build regional sensor to soil property calibrations was tested. ANOCOVA models feature a site-independent (regional) slope coefficient and orchard-specific intercept coefficients. Gamma-ray TC and EC_a_ were used as predictors to map SP and sand, silt, and clay contents. For these regression data a square root transformation was employed to ensure unbiased residuals. The models were evaluated using the coefficient of determination (R^2^), the root mean square error (RMSE), and the mean absolute error (MAE). Models with MAE > 5% were considered not acceptable (Pätzold et al., 2020). The ANOCOVA models were calibrated on all available data (All Data model) and using five soil samples per field (Limited Data model), using the same subsample used for DRIFTS and acid oxalate extractable element analyses. The Limited Data models were evaluated at the left-out locations. Following the methodology of Corwin and Lesch (2014), the regression models were developed using STATISTICA (version 12, StatSoft Inc., Tulsa, OK, USA).

The raw GRS data was investigated to identify any differences in the ratios between TC, K, U, and Th. Differences in these ratios, such as in the Th/K ratio, are often interpreted as differences in parent material clay mineralogy and soil type (Herrmann et al., 2010; Wibowo et al., 2020; Al-Jafar and Al-Jaberi, 2022). At the orchard level, the slope and *r* of the TC linear relations with K, U, and Th, as well as these from K with U and Th, and of U with Th were compared.

Differences in soil concentrations of extractable Fe, Al, Mn, Si, U and Th across study sites were tested with a one-way analysis of variance and the Fisher’s Least Significant Difference *post-hoc* test in STATISTICA. The Th data was left-censored, as three samples had concentrations below limits of detection (0.0004 mg g^-1^). The missing values were estimated dividing the limit of detection by two (Hornung and Reed, 1990).

## 3 Results

### 3.1 Field specific and regional linear relationships between EC_a_, TC, and soil properties

**Table 3** shows the average, median, minimum, maximum, and standard deviation values for the measured soil properties, EC_a_, and TC across the entire dataset, and within the three orchard sites. Lemon Cove was the site with the coarsest soil, 5 locations were classified as Sand, 5 were Loamy Sand, and 10 were Sandy Loam. Riverside had the most homogeneous soil texture, with 19 locations classified as Sandy Loam and 1 as Loam. Strathmore had the most heterogeneous texture: 4 locations were Sandy Loam, 6 were Sandy Clay Loam, 1 was Loam, and 4 were Silt Loam. Most of the soil locations had non-salt affected (EC_e_ < 2 dS/m) soils, 5 locations were slightly saline (2 < EC_e_ < 4 dS/m), of which 3 were in Lemon Cove and 2 were in Riverside.

**Table 3:** Basic statistics for sand, silt, and clay contents; saturated soil extract conductivity (EC_e_); gravimetric water content (GWC) at the time of sampling; saturation percentage (SP), soil apparent electrical conductivity (EC_a_) at the soil sampling locations; and gamma-ray spectrometry (GRS) total counts at the soil sampling locations for the entire dataset and for the three research sites.

	Mean	Median	Minimum	Maximum	Standard Deviation
All sites (n = 55)					
Sand	0.64	0.62	0.30	0.94	0.15
Silt	0.25	0.23	0.04	0.64	0.14
Clay	0.11	0.10	0.02	0.33	0.07
EC_e_ (dS m^-1^)	1.08	0.79	0.30	3.41	0.74
GWC	0.14	0.13	0.04	0.38	0.06
SP	0.32	0.33	0.19	0.52	0.08
EC_a_ (mS m^-1^)	26.97	23.12	14.46	56.66	10.56
GRS Total Counts (cps)	2653.31	2758.54	1584.51	3623.98	744.95
Lemon Cove (n = 20)					
Sand	0.79	0.77	0.64	0.94	0.09
Silt	0.15	0.16	0.04	0.23	0.06
Clay	0.07	0.07	0.02	0.13	0.04
EC_e_ (dS m^-1^)	1.11	0.78	0.39	3.41	0.87
GWC	0.14	0.14	0.11	0.17	0.02
SP	0.24	0.25	0.19	0.29	0.02
EC_a_ (mS m^-1^)	21.31	21.07	18.63	26.73	1.93
GRS Total Counts (cps)	1775.30	1761.19	1584.51	2054.51	117.57
Riverside (n = 20)					
Sand	0.58	0.59	0.45	0.71	0.06
Silt	0.30	0.31	0.20	0.37	0.04
Clay	0.12	0.12	0.07	0.18	0.03
EC_e_ (dS m^-1^)	1.39	1.36	0.58	2.85	0.69
GWC	0.09	0.09	0.04	0.15	0.03
SP	0.35	0.35	0.33	0.39	0.02
EC_a_ (mS m^-1^)	21.87	23.12	14.46	25.96	3.54
GRS Total Counts (cps)	3477.27	3488.88	3302.50	3623.98	93.49
Strathmore (n = 15)					
Sand	0.52	0.53	0.30	0.77	0.15
Silt	0.31	0.20	0.06	0.64	0.20
Clay	0.16	0.17	0.02	0.33	0.09
EC_e_ (dS m^-1^)	0.62	0.51	0.30	1.18	0.28
GWC	0.21	0.19	0.13	0.38	0.07
SP	0.39	0.33	0.27	0.52	0.09
EC_a_ (mS m^-1^)	41.31	35.10	30.47	56.66	10.26
GRS Total Counts (cps)	2725.37	2758.54	2471.73	2992.12	182.13

The number of soil sampling locations (n) is reported.

**Table 4** shows the Pearson *r* coefficients for EC_a_ and TC with the measured soil properties over the entire dataset and for each field. The correlation of EC_a_ with sand was negative and significant (p < 0.05) for all sites and within each orchard. The correlations of TC with sand were significantly negative for the whole dataset, but were non-significant at Lemon Cove, significant and negative at Riverside, and significant and positive at Strathmore. Correlations between silt and EC_a_ and TC were similar to those of sand but with the reverse sign. Clay correlations with EC_a_ were non-significant over the entire dataset and at the Riverside site, they were significant and positive at Lemon Cove and significant and negative at Strathmore. Clay content correlated positively over the entire dataset with TC, but no significant correlations emerged at the single sites. Notably, clay content showed a weak significant correlation with sand content (*r* = -0.43) over the entire dataset, but no other significant correlation with other soil properties. The clay-sand correlations were strongly negative at Lemon Cove (*r* = -0.91) and Riverside (*r* = -0.80), but non-significant at Strathmore. The clay-silt correlations were positive at Lemon Cove (*r* = 0.77) and Riverside (*r* = 0.51), but negative at Strathmore (*r* = -0.72). Over the entire dataset, the relationships between SP and sand were strong and negative (*r* =-0.86), but non-significant with clay. Over the entire dataset, TC had a non-significant correlation with EC_a_. The only significant relationship was observed between TC and EC_a_ at the Strathmore site with *r* = -0.89.

**Table 4:** Pearson correlation coefficients for soil apparent electrical conductivity and gamma-ray total counts with sand, silt, and clay contents; saturated soil extract conductivity (EC_e_); gravimetric water content (GWC) at the time of sampling; and saturation percentage (SP).

	All sites	Lemon Cove	Riverside	Strathmore
Apparent Electrical Conductivity				
Sand	**-0.70**	**-0.73**	**-0.55**	**-0.92**
Silt	**0.65**	**0.67**	**0.59**	**0.94**
Clay	0.24	**0.72**	0.32	**-0.56**
EC_e_	**-0.35**	0.22	-0.34	-0.12
GWC	**0.81**	0.10	0.41	**0.80**
SP	**0.72**	0.08	-0.03	**0.94**
Gamma-Ray Total Counts				
Sand	**-0.55**	0.11	**-0.58**	**0.88**
Silt	**0.43**	-0.13	**0.63**	**-0.83**
Clay	**0.37**	-0.05	0.32	0.40
EC_e_	0.14	0.17	-0.27	0.02
GWC	**-0.32**	0.22	-0.07	**-0.68**
SP	**0.54**	**-0.47**	-0.23	**-0.91**

Bold and red coefficients were significant at the p<0.05 level.

**Table 5** reports the ANOCOVA regressions for sand, silt, and clay content, and SP with EC_a_ as the predictor. The ANOCOVA regression models were developed on square root transformed data. ANOCOVA regression assumptions were met. The goodness-of-fit metrics (R^2^, RMSE, and MAE) were calculated for the back-transformed data. For all models, except the one predicting clay content, the ANOCOVA slopes in the All Data models and Limited Data models had slope values with overlapping standard errors. The calibration of the All Data and Limited Data models were significant at the p<0.05 level. The model calibration errors were low, all calibration MAEs were acceptably low (i.e., MAE < 0.05). The independent evaluations of the Limited Data models had MAE <0.06. The calibration RMSE values were under 0.06 in the All Data and Limited Data calibrations. The RMSE for the independent evaluations of the Limited Data models were 0.07 for sand, silt, and SP and 0.073 for clay content.

**Table 5:** The analysis of covariance regression statistics for soil apparent electrical conductivity (independent variable) and sand, silt, and clay content, and saturation percentage (SP) (dependent variables).

Dependent variable	Slope (standard error)*		R^2^	L.D.	Ind. Eval.	RMSE	L.D.	Ind. Eval.	MAE	L.D.	Ind. Eval.
	A.D.	L.D.	A.D.			A.D.			A.D.		
Sand	-0.112(0.011)	-0.12(0.014)	0.84	0.94	0.80	0.060	0.040	0.066	0.049	0.030	0.055
Silt	0.183(0.019)	0.21(0.022)	0.81	0.97	0.73	0.059	0.030	0.069	0.048	0.024	0.055
Clay	-0.05(0.023)	-0.13(0.031)	0.38	0.65	0.10	0.052	0.037	0.073	0.039	0.027	0.054
SP	0.061(0.008)	0.067(0.01)	0.84	0.93	0.81	0.031	0.020	0.035	0.025	0.016	0.028

*Regression slopes and standard errors are for the square-root-transformed data.

The regression slope (standard error in parenthesis) for the All Data (A.D.) and Limited Data (L.D.) models are reported. The goodness of fit for the A.D. and L.D. models and the independent evaluation of the L.D. models are reported: the coefficient of determination (R^2^), the root mean square error (RMSE), and the mean absolute error (MAE).

The ANOCOVA approach could not be employed to estimate any of the texture-related soil properties using TC. The relationships between the sensor and each soil property could not be represented with a single slope across the entire dataset.

### 3.2 Differences in soil characteristics across the three sites

**Figure 3:**
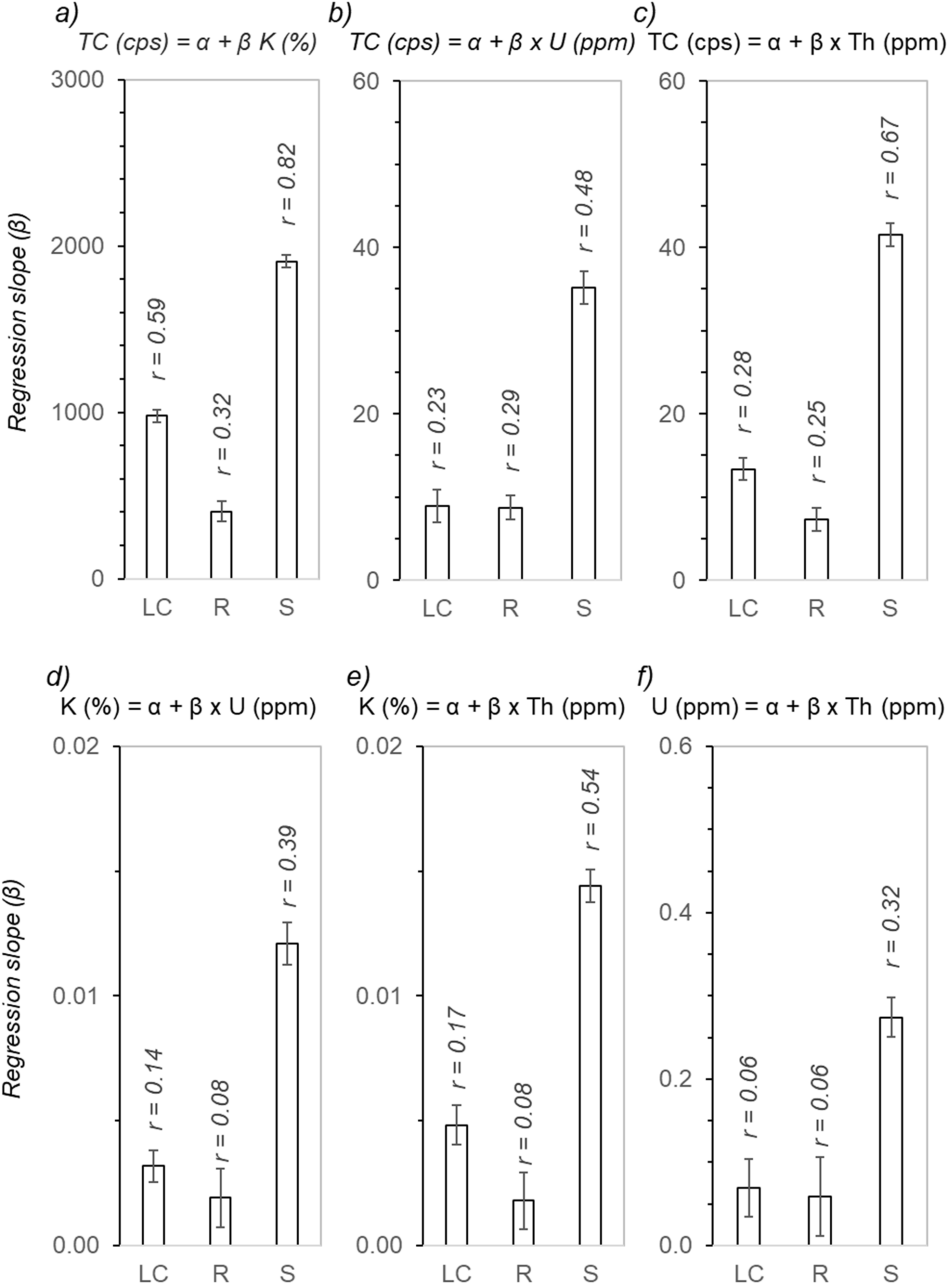
Regression slope coefficients (bars are the 5_th_ to 95_th_ confidence interval for the coefficients) and Pearson correlation coefficients (*r*) for relationships between gamma-ray spectrometry total counts (TC), potassium (K), uranium (U), and thorium (Th). Each quadrant compares regression slopes at the three research sites, Lemon Cove (LC), Riverside (R), and Strathmore (S), for the simple linear regression model (α is the regression intercept) specified at the top of each quadrant: TC as a function of **(a)** K, **(b)** U, and **(c)** Th; K as a function of **(d)** U, and **(e)** Th; and **(f)** U as a function of Th.

**Figure 3** reports the slope and the Pearson *r* for the linear relationships for all combinations between TC, K, U, and Th from the raw (i.e., non-kriged) sensor datasets. The sample sizes for the three datasets were large (see **Table 1**). The *r* values were all significant at the p<0.05 level. For all considered linear relationships, the *r* values were visibly higher at Strathmore than at the other two sites. Similarly, the slope coefficient values for all relationships were remarkably higher at Strathmore than at the other two sites. In **Figure 3**, the error bars represent the 5^th^ to 95^th^ confidence interval for the regression slopes. Notably, the slope intervals at Lemon Cove and Riverside overlapped (i.e., not significantly different) for the TC (dependent) and U (independent) relationship (**Figure 3b**), for the K (dependent) and U (independent) relationship (**Figure 3d**), and for the U (dependent) and Th (independent) relationship (**Figure 3f**).

**Figure 4:**
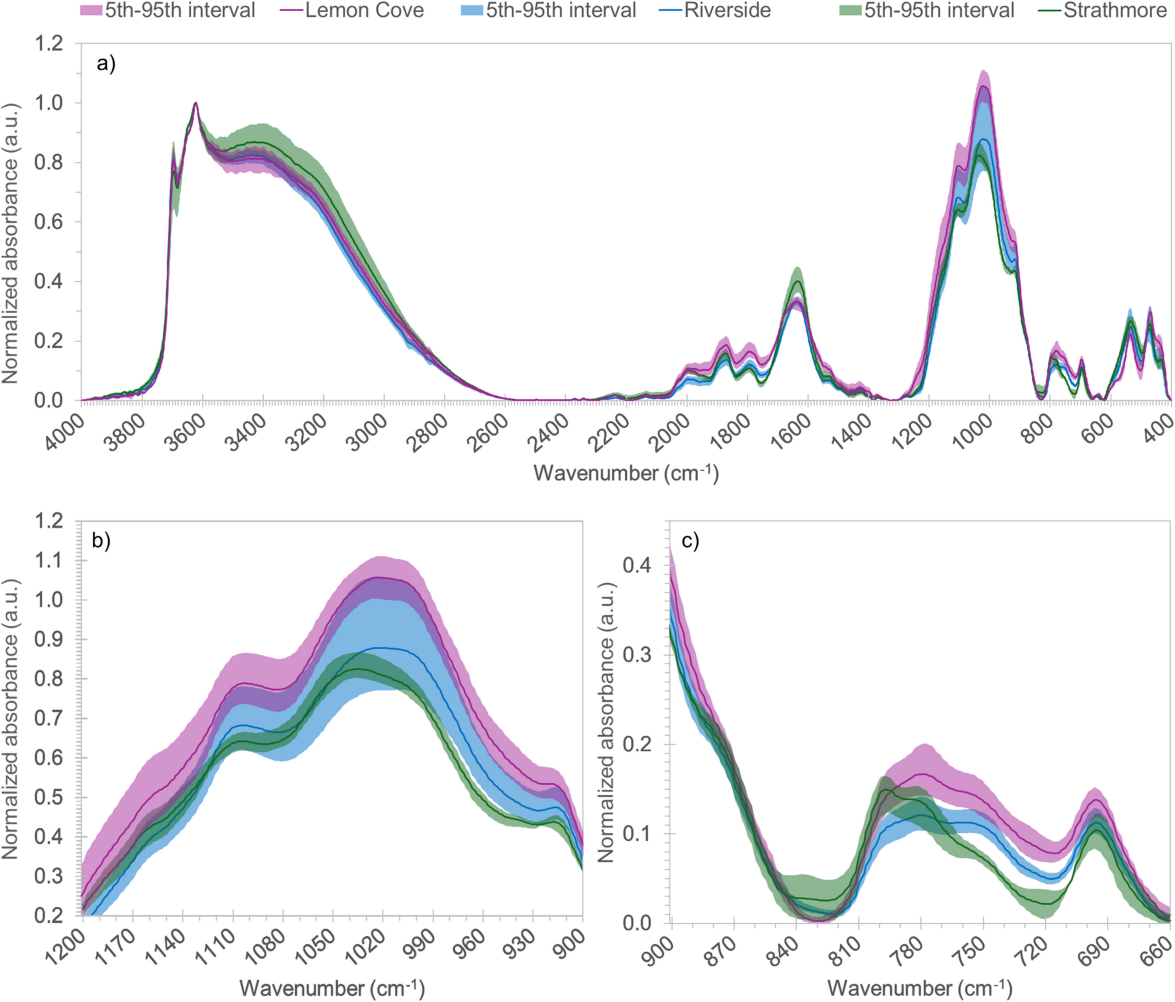
Diffuse reflectance infrared Fourier transform spectra for soil samples from the three sites. The average spectral signature for each site (solid lines) and the 5th to 95th interval (shaded areas) are depicted. The 4000-400 cm-1 spectra are shown in quadrant **(a)**. Quadrant **(b)** shows the specra between 1200 and 900 cm-1. Quadrant **(c)** shows the specra between 900 and 660 cm-1.

Across all sites, DRIFTS spectra showed prominent aluminosilicate clay mineral vibrations which informed the nature of clay minerals present (**Figure 4**). This includes features generally related to structural -OH (≈3697 and 3623 cm^-1^), Si-O (≈1100, 1035-1015, 790-430 cm^-1^), octahedral Al-Al-OH (≈915 cm^-1^), Al-O-Si (≈535 cm^-1^) and clay-associated water (≈3400 and 1638 cm^-1^) vibrations present across all sites (Madejová and Komadel, 2001; Madejová, 2003; Petit, 2006). The -OH stretching mode at 3697 cm^-1^ was indicative of kaolinite clay present in soils, while peaks around 3620 and 3400 cm^-1^ have been identified previously as being characteristic of montmorillonite, suggesting the presence of these two clay minerals across sites (Khang et al., 2016). There were subtle differences between sites that suggested some differences in mineralogy between sites. This was primarily observed for the Strathmore soils, which showed some vibrational features either distinct or more pronounced when compared to the Lemon Cove and Riverside soils. This includes a pronounced peak at 798 cm^-1^, a more pronounced shoulder at 878 cm^-1^, a reduction of the 750 cm^-1^ feature, and a primary Si-O peak at 1035 cm^-1^ in Strathmore compared with 1019 cm^-1^ in Lemon Cove and Riverside soils. These differences could have potentially resulted from changes in clay octahedral sheet substitution for a given clay mineral type, which could have enhanced absorbance near 878 cm^-1^ as well as impacted the position of the primary Si-O stretching peak near ≈1030-1015 cm^-1^ (Madejová and Komadel, 2001; Madejová, 2003). Differences in mineral composition between Strathmore and the Lemon Cove and Riverside sites, including aluminosilicate and oxide species, could also have resulted in different absorbances across sites as several different minerals have DRIFTS features in this range (e.g., goethite, silicon oxide minerals) (Madejová, 2003; Parikh et al., 2014). All of these potential influences indicated a Strathmore soil mineralogy distinct from Lemon Cove and Riverside.

Poorly crystalline forms of Fe, Al, Mn and Si (i.e., organically bound and amorphous mineral species) varied between sites (**Table 6**). Lemon Cove soils had the highest average concentration of acid ammonium oxalate extractable Fe and was significantly higher than Strathmore, with Riverside being statistically similar to both. Strathmore soils had significantly higher average extractable concentrations of Al and Mn than Lemon Cove, with Riverside comparable to both sites. Extractable Si was highest for Riverside, with Lemon Cove significantly lower. U associated with these forms was highest in Lemon Cove, followed by Riverside and then Strathmore, with Strathmore being significantly lower than Lemon Cove. Th was significantly higher in Lemon Cove compared to Strathmore, which had detectable Th in only 2 of the 5 soils analyzed, with Riverside not significantly different from either. Across all sites, U and Th associated with extractable elements showed a positive correlation with extractable Fe phases, while extractable Al, Mn and Si had a negative relationship with associated U and Th (**Table 7**).

**Table 6:** Fe, Mn, Al and Si in acid oxalate extractable solid phases along with Th and U associated with these phases.

Site	Fe_ao_	Mn_ao_	Al_ao_	Si_ao_	Th_ao_	U_ao_
(mg g^-1^ soil)						
Lemon Cove	2.57 a	0.13 c	0.45 b	0.29 b	0.011 a	0.027 a
Riverside	1.01 b	0.27 b	0.59 b	0.51 a	0.0061 ab	0.015 b
Strathmore	0.76 b	0.44 a	0.84 a	0.41 a	0.0016 b	0.011 b

Denoted statistical significance (p<0.05 level) was determined through one way Analysis of Variance and the Fisher’s Least Significant Difference as a *post-hoc* test.

**Table 7:** Pearson correlation coefficients for soil concentrations of extractable Th and U with the extractable Fe, Mn, Al, and Si.

	Fe	Mn	Al	Si
Th	**0.60**	**-0.69**	**-0.62**	-0.48
U	**0.98**	**-0.81**	**-0.62**	**-0.57**

Bold and red coefficients were significant at the p<0.05 level.

## 4 Discussion

Arguably, on-the-go soil sensors should reliably correlate with the target soil properties that they developed to justify their broad and consistent use in high-resolution soil mapping. On-the-go sensors measuring EC_a_ capture a complex process which is influenced by many soil properties such as water content, salinity, and soil texture. These complex interactions contributing to EC_a_ have been studied in agricultural soils for many decades (Rhoades et al., 1976, 1989; Corwin and Lesch, 2003; 2005a), and are fairly well understood: e.g., higher clay content, water content, and salinity contribute to higher EC_a_. Moreover, it is often the case that the spatial patterns of the soil properties influencing EC_a_ are locally correlated at the field scale, e.g., the soil physical properties and terrain drive the spatial variability of soil moisture and salinity, making statistical EC_a_-to-soil-property calibrations at the field-scale fairly straightforward (Corwin and Scudiero, 2016). Because of the complex interactions between soil properties influencing EC_a_, unexpected soil relationships are sometimes reported. For example, Scudiero et al. (2016) reported a negative relationship between EC_a_ and clay content from fields in shallow soils on hilly landscape in Colorado, USA. The GRS as reviewed by Mahmood et al. (2013) measures a more straightforward process: the emissions of gamma radiation from radionucleotides naturally occurring in soils and rocks (mainly from ^40^K, ^238^U, and ^232^Th) and from anthropogenic ^137^Cs. The overall TC are expected to be lower in sandy soils than in finer soils (Mahmood et al., 2013; Reinhardt and Herrmann, 2019); and others, and as supported by the global Pearson correlation coefficients observed in this study (**Table 4**). Unfortunately, at the field-scale, TC relationships with soil texture can be erratic as shown by Pätzold et al. (2020) in Germany, by Maxton and Lund (2020) in the US Midwest, and as observed in this California citrus study (**Table 4**).

Despite the potential of observing unexpected correlations between EC_a_ and GRS with texture-related field properties, we observed significant relationships between most target soil properties and sites using either of the sensors (**Table 4**). Moreover, as discussed by Scudiero et al. (2024a), data fusion (e.g., principal component analysis, multiple regression) between the two sensors can be a means to obtain very accurate soil texture maps. Unfortunately, even when leveraging the spatial information from on-the-go sensing in spatial models (Reyes et al., 2018) and/or directing the soil sampling to calibrate ordinary least square models (Lesch, 2005), the amount of ground-truth data needed to develop accurate field-scale maps is too high (e.g., dozens of soil samples per field). The burden and the cost associated with the collection of ground-truth data from numerous locations within each field is a major bottleneck for the widespread application of on-the-go sensing for quantitative and accurate soil texture mapping.

Beyond the field-scale, the potential for developing regional site-independent models that would allow predicting soil properties at new sites without the need for collecting local ground-truth data has been investigated by many scientist in the past decades. Sudduth et al. (2005) investigated the use of EC_a_ from different sensors to map texture and cation exchange capacity across six US Midwest states. Their study sites from six states included soils of differing parent material, weathering, levels of organic matter content, and agricultural management. They observed R^2^ values ≥ 0.55 for the two soil properties across their entire dataset. Although encouraging, their models were not sufficiently accurate to predict field-scale texture for precision agriculture. For GRS, Heggemann et al. (2017) developed a site-independent model to predict soil texture using data collected from ten agricultural fields in Germany across sites with diverse mineralogy and parent material. They developed site-independent support vector machines models that yielded R^2^ of 0.96 (sand), 0.93 (silt), and 0.78 (clay), and with MAE values < 0.04. However, Pätzold et al. (2020) evaluated the model by Heggemann et al. (2017) at independent fields in Germany. They observed that the “model was not generally capable of predicting soil texture at sites that were not adequately represented in the calibration set”.

ANOCOVA regression as an alternative approach to universal models has been widely discussed for EC_a_-to-salinity calibrations (Corwin and Lesch, 2014, 2017; Scudiero et al., 2017). Harvey and Morgan (2009) discussed the potential of ANOCOVA for texture mapping on three fields in Texas, USA, and observed prediction errors below 4% for clay. In this study, we report calibration MAE values < 0.05 for ANOCOVA EC_a_-texture predictions (**Table 5**). Previous research concluded that ANOCOVA may be a means to reducing field-scale soil samples (Corwin and Lesch, 2014; Scudiero et al., 2017). In particular, the regression slope common for all fields is calibrated over the entire available dataset, whereas only three or more samples per field may be needed to estimate the field-specific intercept. For the first time, this research shows that minimal data (n=5 per field) can be used to calibrate accurate ANOCOVA EC_a_-texture regression with very low calibration and independent evaluation errors (**Table 5**). Generating high-resolution, accurate soil maps using as few as five ground-truth sites per agricultural field may benefit practitioners seeking to decrease the costs related to soil sampling and laboratory analyses.

ANOCOVA regression was shown in this research to be a very powerful tool to map texture with limited data, but it relies on postulating the slope between the sensor and the target soil property to be stable over multiple fields. This was not the case for GRS-TC making ANOCOVA regression modeling not feasible. Over the three fields, the TC showed expected (e.g., positive *r* with clay) and unexpected (e.g., negative *r* with clay) with the target soil texture properties. These inconsistencies make GRS unsuitable for texture mapping in California citrus orchards using ANOCOVA. Field-specific modeling may be needed instead. Mahmood et al. (2013) and Pätzold et al. (2020) indicated that differences in parent material and clay mineralogy may be responsible for contrasting GRS and texture relationships over multiple fields. If fields with unexpected GRS-texture relationships could be identified from raw GRS data or using available landscape-scale soil maps, then soil scientists may decide whether to include such fields in ANOCOVA models or to calibrate field-specific models (which require larger ground-truthing). Moreover, reliable classification of the expected nature of the GRS-texture relationship may enable the calibration of reliable site-independent models (Pätzold et al., 2020). For this reason, soils from the three citrus sites were analyzed using DRIFTS, extraction of active mineral phases, and available USDA soil maps (Beaudette and O’geen, 2009) in relationship to the raw GRS TC, K, Th, and U observed at the three sites.

Mineralogical analysis through DRIFTS and extraction of active Fe, Al, and Mn oxides highlighted notable differences in soil clay fraction mineralogy between sites. The aluminosilicate and active oxide phases probed here both represent prevalent, reactive and high surface area minerals in soil clay size fractions that are known to retetain several elements in soils, including K, Th, and U (Mcbride, 1994; Duff et al., 2002; Bachmaf and Merkel, 2011; Hongxia et al., 2016; Dublet et al., 2017; Wang et al., 2021). As such, variation in this mineralogy between sites may translate to different affinities of these isotopes for the clay fraction of these soils. In the case of the soil minerals probed by DRIFTS, there were apparent differences in clay substitution, clay composition and/or oxide chemistry between the Strathmore site and Lemon Cove and Riverside soils. These factors could have impacted surface hydroxyl group availability (e.g., 1:1 vs. 2:1 clay), cation exchange capacity and exchange selectivity, which may greatly impact mineral retention of ions, including Th and U (Duff et al., 2002; Bachmaf and Merkel, 2011; Hongxia et al., 2016; Wang et al., 2017). In addition to the distinct aluminosilicate mineralogy at the Strathmore site, differences in extractable Fe, Al, Mn, and Si provide further insight into the deviation in GRS-texture relationship at the Strathmore site. An observation apparent in our data is the role of poorly-crystalline and amorphous Fe species in U and Th retention across sites, in alignment with previous studies showing the strong association of these Fe species for U and Th through adsorption and co-precipitation (Duff et al., 2002; Dublet et al., 2017; Li et al., 2019; Wang et al., 2021). Interestingly, poorly-crystalline Al, Mn, and Si forms showed a weak inverse relationship with extracted U and Th, indicating they did not associate to the same extent as with Fe. Provided the relatively lower proportion of Fe in total extracted poorly-crystalline mineral phases at the Strathmore site compared to Lemon Cove and Riverside, it is conceivable that this reduced the affinity of Th and U for fine-grained active oxides in Strathmore. This could, in turn, weaken or alter the relationship between soil clay content and GRS through lower radionuclide retention. While these results overall indicate that mineralogy may have played a role in observed inconsistencies in assessment of soil texture across sites with GRS, more detailed mineralogical analyses (e.g., X-ray diffraction “XRD”), extended X-ray absorption fine structure “EXAFS” spectroscopy) and isotope retention studies of isolated textural fractions across sites would further explore this relationship.

The mineralogical results align with the previous suggestion that differences in clay fraction mineralogy may result in contrasting trends of GRS and clay determination between field sites (Mahmood et al., 2013; Pätzold et al., 2020). This suggests that fundamental knowledge of site mineralogy may inform the applicability of GRS for soil textural determination between sites. This could relate further to variable environmental factors that may impact mineralogy of soil clay fractions, such as parent material, drainage and climate (Mcbride, 1994). For example, formation and dissolution of poorly crystalline Fe species related to Th and U retention across the Lemon Cove, Riverside and Strathmore sites are notably sensitive to soil redox fluctuations that may in turn impact radionuclide retention (Duff et al., 2002; Winkler et al., 2018). Knowing in advance the pedological properties of a soil may therefore provide some indication of what type of GRS defined textural relationships could be expected for a given site. Defining these relationships with respect to widely available soil survey data may also represent a future area for exploration with substantial implications for GRS application at regional scales.

## 5 Conclusions

As smart fertilizer and water management practices become increasingly important for California’s specialty crops, driven by factors such as resource scarcity, rising costs, and regulations, the need for accurate, high-resolution soil maps will grow. Geospatial sensors, such as soil apparent electrical conductivity (EC_a_) and gamma-ray spectrometry (GRS), were confirmed in this study as reliable tools for field-scale soil mapping of particle size fraction, based on data from three citrus orchards in California. Model-based sampling schemes, such as the response surface sampling design used here, enable the creation of accurate soil maps using a relatively small set of ground-truth soil samples. Multi-field modeling using universal, site-independent models may not be feasible due to unknown secondary influences on sensor measurements at the individual field level. For EC_a_, locally adjusted analysis of covariance (ANOCOVA) regressions modeled particle size fractions with high accuracy. Notably, the ANOCOVA regressions can be calibrated using limited (n=5 per field) data. This novel insight marks a step forward in making high-resolution mapping affordable for practitioners and their clientele. More research is needed to understand how ANOCOVA models that use minimal soil data can be developed, calibrated, evaluated, and improved over time (e.g., when any new field is added to a preexisting dataset).

In one of the orchards (the Strathmore orchard), GRS exhibited an unexpected negative correlation with clay content, making the use of ANOCOVA for GRS-texture regressions not possible. Although such relationships have been reported previously, the causes behind them remain poorly understood. To investigate this further, GRS ratios, diffuse reflectance infrared Fourier-transform spectroscopy (DRIFTS), and acid ammonium oxalate extractable elements were analyzed at all three sites. The Strathmore orchard displayed unique GRS ratios, DRIFTS, and acid ammonium oxalate extractable element profiles compared to the other sites. These novel insights may guide future research and help predict whether positive (expected) or unexpected GRS-clay content relationships are likely to occur at a given field, based either on raw GRS data or geochemical information. Such a-priori knowledge could inform soil scientists’ decisions about ground-truthing efforts (e.g., allocating additional resources to increase the number of soil samples for an accurate map) or be used as an additional predictor in regional models, such as support vector machines or random forest regression models. Here, the odd GRS-texture relationships were observed in one site only, which had fewer sampling locations than the other two sites. Further research is needed to identify other sites showing unexpected GSR-texture relationships to understand commonalities amongst these and contrasting features compared to sites where GSR performs as expected.

## Data Availability

The raw data supporting the conclusions of this article will be made available by the authors, without undue reservation.
